# Recent Advances in Biomolecular Recognition

**DOI:** 10.3390/ijms24098310

**Published:** 2023-05-05

**Authors:** Qiang Zhu, Ray Luo

**Affiliations:** Departments of Molecular Biology and Biochemistry, Chemical and Biomolecular Engineering, Materials Science and Engineering, and Biomedical Engineering, University of California, Irvine, CA 92697, USA

Living cells are extremely complicated systems and composed of hundreds of thousands of diverse biomolecules, such as proteins, nucleic acids, and carbohydrates. They interact with each other to maintain a wide range of cellular functions, including cell signaling, enzymatic reactions, DNA replication and transcription. These interactions are collectively termed biomolecular recognition.

Recent research efforts in biomolecular recognition have mostly focused on the following two aspects: specificity and affinity. Specificity ensures that biological molecules can fulfill their intended function without interfering with other processes. Affinity ensures that molecular interaction is stable enough for biological functions. Many factors can affect specificity and affinity, such as the shape and size of the binding site, the charge state, and the nature of the chemical interactions.

Understanding the principles of biomolecular recognition is of great significance in the development of new drugs and therapies. In this Special Issue entitled “Recent Advances in Biomolecular Recognition” of the *International Journal of Molecular Sciences*, both wet- and dry-lab researchers contribute a total of five original articles. These research articles cover a broad range of fields, such as the prediction of protein–protein interaction site (PPIs), elucidation of the roles of electrostatic interactions in the capsid assembly of giant viruses and human respiratory syncytial virus M${}_{2-1}$, and the kinetics of drug–DNA interactions.

Identifying protein–protien interaction sites (PPIs) through experimental methods is a challenging task, which is not only time-consuming and laborious but also error-prone, as there is a high likelihood of both false positives and false negatives. Deng et al. [1] proposed two computational models based on the extreme gradient boosting (XGBoost) algorithm to predict PPIs. Good performance and accuracy were achieved when unbalanced data sampling methods, namely, repetitive nearest neighbor (RENN) and instance hardness threshold (IHT), were applied. A better prediction performance, such as an 80.71% accuracy rate and 0.614 of Matthews correlation coefficient (MCC) was achieved when IHT was combined with XGBoost.

Xian et al. [2] implemented a multi-scale method to investigate the interactions within *Paramecium bursaria* Chlorella virus-1 (PBCV-1) capsid building units. Three binding modes were detected at the icosahedral two-fold region of PBCV-1 capsid. With the help of molecular mechanics with Poisson–Boltzmann and surface area solvation (MM/PBSA) and Delphi, they analyzed the binding free energies and electrostatic binding force between capsomers and concluded that electrostatic interactions are crucial in viral capsid assembly and guide the capsomers to form a stable assembly along a favored distance and orientation. To capture the capsid assembly process, a capsomer structure manipulation package was developed.

Beniaminov et al. [3] investigated the interactions between antibiotic olivomycin A (OA) and the minor groove of the G/C rich double-stranded DNA. With the help of electrophoretic mobility gel shift assay (EMSA), they screened the complete set of tetranucleotide G/C sites and revealed that the complex formed between the binding site of central dinucleotide CG and OA is kinetically less stable than dinucleotides GG or GC. Although a similar affinity of the antibiotic and diverse G/C DNA sequences was detected using fluorescence, circular dichroism and isothermal titration calorimetry, this discrepancy could be resolved by the dissociation kinetic of the drug–DNA complex. The differential kinetics of OA–DNA interactions were later demonstrated to be functional and relevant in an in vitro transcription assay.

Piva et al. [4] employed both experimental techniques and computational tools and disclosed the interactions between human respiratory syncytial virus (hRSV) M${}_{2-1}$ and flavanones such as hesperetin (HST) and hesperidin (HSD). Through saturation transfer difference nuclear magnetic resonance spectroscopy (STD-NMR) analysis, they found that both HST and HSD bind to M${}_{2-1}$ with aromatic rings positioned on the target protein binding site. With the help of fluorescence-quenching measurements, a stronger interaction affinity was observed between HST and M${}_{2-1}$ than that of HSD. A detailed thermodynamic analysis further suggested that hydrogen bonds and van der Waals interactions play significant roles in the complex stabilization. Molecular dynamics simulations indicate that a possible interaction site is the adenosine monophosphate (AMP)-binding site in M${}_{2-1}$, which is in line with the experimental results.

Zazeri et al. [5] carried out several spectroscopic techniques and computational techniques to characterize the interactions between rat serum albumin (RSA) and piperine. The binding constant and number of ligands were estimated to be $3.9\times 10^{4}$ M${}^{-1}$ at 288 K using the Stern–Volmer model and three, respectively, based on the spectroscopic results. The estimated Gibbs free energy (ΔG=−25 kJ/mol) indicates that the interaction is spontaneous. Energy decomposition analysis shows that the main contribution mainly derives from the enthalpic term and the stability of the complex is maintained by hydrogen bonds.

Advances in biophysical techniques including X-ray crystallgraphy, nuclear magetic resonace spectroscopy, and cryo-electron microscopy have greatly expanded our ability to investigate biomolecular recognition at the atomic level. In addition, a significant portion of the theoretical foundation and computational methods has been established and developed. This is a great chance to combine both experimental and theoretical techniques to deliver important insights into the mechanisms of biomolcular recognition and open up new opportunities for drug discovery and design.
